# Engaging a Chemical Disaster Community: Lessons from Graniteville

**DOI:** 10.3390/ijerph110605684

**Published:** 2014-05-27

**Authors:** Winston Abara, Sacoby Wilson, John Vena, Louisiana Sanders, Tina Bevington, Joan M. Culley, Lucy Annang, Laura Dalemarre, Erik Svendsen

**Affiliations:** 1 Department of Community Health and Preventive Medicine, Satcher Health Leadership Institute, Morehouse School of Medicine, Atlanta, GA 30310, USA; E-Mail: wabara@msm.edu; 2 Maryland Institute for Applied Environmental Health, University of Maryland, College Park, MD 20742, USA; 3 Department of Public Health Sciences, Medical University of South Carolina, Charleston, SC 29425, USA; E-Mail: vena@musc.edu; 4 Graniteville Community Coalition, Graniteville, SC 29829, USA; E-Mail: louwright@earthlink.net; 5 GRACE Study Center, Graniteville, SC 29829, USA; E-Mail: bevington1959@hotmail.com; 6 College of Nursing, University of South Carolina, Columbia, SC 29208, USA; E-Mail: jculley@mailbox.sc.edu; 7 Department of Health promotion, Education, and Behavior, University of South Carolina, Columbia, SC 29208, USA; E-Mail: lannang@mailbox.sc.edu; 8 Department of Global Environmental Health Sciences, Tulane University, New Orleans, LA 70112, USA, E-Mail: esvendse@tulane.edu

**Keywords:** community engagement, community health partnerships, chemical disasters, community-based participatory service, community-based participatory research, environmental health, sustainability

## Abstract

Community engagement remains a primary objective of public health practice. While this approach has been adopted with success in response to many community health issues, it is rarely adopted in chemical disaster response. Empirical research suggests that management of chemical disasters focuses on the emergency response with almost no community engagement for long-term recovery. Graniteville, an unincorporated and medically underserved community in South Carolina was the site of one of the largest chlorine exposures by a general US population. Following the immediate response, we sought community participation and partnered with community stakeholders and representatives in order to address community-identified health and environmental concerns. Subsequently, we engaged the community through regular town hall meetings, harnessing community capacity, forming coalitions with existing local assets like churches, schools, health centers, and businesses, and hosting community-wide events like health picnics and screenings. Information obtained from these events through discussions, interviews, and surveys facilitated focused public health service which eventually transitioned to community-driven public health research. Specific outcomes of the community engagement efforts and steps taken to ensure sustainability of these efforts and outcomes will be discussed.

## 1. Introduction

Disasters can be natural or technological events that produce considerable physical, social, political and economic disruptions to a community’s functional system often resulting in severe damage and death [1,2,3]. Natural disasters include earthquakes, floods, tornadoes, and tsunamis [4] while technological disasters include the unexpected release of noxious materials like chemical or nuclear explosions, radiological or biological emissions, oil spills and mining accidents [5,6]. Unlike natural disasters, technological disasters are usually associated with flaws, defects, and negligence of man-made infrastructure and processes, and are also more likely to occur without warning [6]. An example of this includes the leaching of noxious materials from a leaking storage area with negative environmental consequences. Technological disasters may also be due to the inadvertent secondary or indirect effects of otherwise safe processes and systems which result in harm to people, their surroundings, and the environment [7]. A peculiar characteristic of this type of technological disaster is the uncertainty that a disaster has occurred because of delayed evidence of harmful consequences over a period of time [7]. The destruction of the habitat that occurs years after “safe” mountaintop mining extraction is an example of this type of technological disaster [7].

The last century and the early parts of this century have witnessed an increase in the occurrence of technological disasters, especially chemical disasters [8]. While some of these disasters have occurred following natural disasters, such as the radiation disaster after the Japanese tsunami [9], others have occurred as a direct result of human activities or human error; the Deepwater Horizon oil spill [10], Bhopal methylisocyanate release [11], Chernobyl radiation release [12], and Seveso dioxin release [13] are examples. This increase in the incidence of chemical disasters has been attributed in part to increased industrial and technological growth [13]. With industrialization and technological advancement, there has been an accompanying increase in the use of alternative and sometimes potentially dangerous sources of energy in order to meet production and consumer demands. In addition, there may be increased utilization of chemicals as ingredients, or their generation as a byproduct in the manufacturing and production process. Furthermore, the increased use, storage and disposal of these chemical compounds and their byproducts ensure that they have to be constantly transported [14]. The complexity and multiplicity of the interactions involved in these processes increases the likelihood of the occurrence of chemical disasters despite precautionary measures that may be in place to prevent the disaster [14].

The consequences of chemical disasters may be long lasting, wide-ranging [15] and are largely dependent on the degree of exposure [15,16]. Exposure assessment is therefore crucial as it determines interventions that alleviate the implications of chemical exposure [16]. Vallero and Lioy describe five chemical exposure stages (rescue, recovery, reentry, reconstruction, and rehabitation) following a disaster [16]. The exposure that occurs during the rescue stage is acute and the goal of exposure measurement is to protect first responders and to understand the magnitude of injury or death that may have occurred. The recovery stage focuses on exposure assessment that aims to evaluate long-term public health consequences of the chemical disaster and further minimize the exposure of first responders. The next stage is the reentry stage and exposure assessment centers on creating a safe environment for human activities to be normalized and this can range from a days to months. Exposure measurements are also important during reconstruction (building or rebuilding) and rehabitation (long-term occupation) stages to mitigate chemical exposure and long-term health and environmental impacts.

For example, the release of noxious chemicals may contaminate water, air and soil, disrupting routine social and economic activities and delaying the immediate re-occupation of the community pending a chemical hazard risk assessment [15]. Additionally, health effects from exposure to these noxious chemicals may manifest as both acute and chronic physical or psychological disorders such as cancers [17,18], congenital anomalies [19], asthma [17], emphysema [17], chronic obstructive pulmonary disease [17], cardiovascular outcomes [17], personality/psychiatric disorders [20], endocrine [17], and immune system dysfunction [17,20]. These effects may be magnified in communities already struggling with underlying economic and healthcare vulnerabilities that impair disaster response and recovery [21,22]. In essence, the impact of a chemical disaster is multi-faceted, does not solely depend on its magnitude but also on the preparedness level of the community to mitigate the immediate and long-term effects of chemical disasters.

## 2. Current Disaster & Health Response in the United States

Current disaster response in the US is guided by the National Response Framework developed by the Federal Emergency Management Agency (FEMA) [23]. The framework includes all requisite actions and plans that should occur in the event of a disaster. It also goes on to outline strategies to save lives, protect property and the environment, meeting basic needs, and support short-term recovery. The framework emphasizes the synergistic roles of various agencies and partners at the local, tribal, state and federal levels to ensure effective and successful disaster response [23]. It describes effective disaster response in three phases: (1) preparedness; (2) response; and (3) recovery [23].

Preparedness is the first phase and requires that disaster relief mechanisms are in place prior to the occurrence of a disaster. This involves disaster mitigation planning, pre-disaster recovery planning, conducting disaster preparedness exercises, and developing disaster plan protocols in anticipation of a disaster while simultaneously evaluating and improving existing disaster response infrastructure [23]. It requires that the police, fire and emergency services, local businesses and community residents, ensure that a community is constantly prepared to respond to a disaster whenever and however it occurs while improving and making adjustments to its disaster response strategies [23].

The response phase occurs immediately following a disaster and it involves two concurrent processes—emergency response and disaster assessment [23]. The emergency response is spearheaded by local emergency departments such as law enforcement, fire and hazardous materials response units, and emergency medical staff. The primary focus of this response is to save lives and property, control damage, preserve disaster site, and evacuate affected individuals to hospitals and safe areas if need be. Simultaneously, a disaster assessment is conducted which determines the geographical area and population impacted, and the degree to which they, as well as community infrastructure and operations are impacted. This is driven by environmental health specialists, engineers, public health practitioners, disaster epidemiologists, health administrators and other professionals. At the same time, resources, assets, and personnel are called upon at the state and possibly federal level and synchronized to ensure effective response. During this phase, prompt exchange of information regarding disaster severity, emergency resource centers, and evacuation plans, between partner agencies and the public is crucial.

Additionally, the needs of the impacted population such as food, water, shelter and clothing, especially among vulnerable groups (e.g., elderly and children), must be identified and met. Other aid in the form of mental and behavioral health services support during this period must be provided. Health surveillance systems to monitor and detect health outbreaks that may be a direct result of or be a consequence of the disaster should also be activated.

Once the response phase and its activities cease, the recovery phase commences [23]. This phase focuses on returning the disaster-impacted community to normalcy (*i.e.*, pre-disaster conditions). Emphasis is on assisting individuals and households in meeting basic needs like food, clothing and shelter. This phase also centers on the provision of temporary but essential utility and infrastructure such as road, housing, power and water delivery infrastructure, medical and public health services, and restoring basic community services and functions [23]. The phase can last from days to months and lays the groundwork towards social, political, and economic restoration and functioning such as the re-opening of schools, government offices, social centers, and recreational attractions.

While most of these actions taken towards community self-sufficiency are in the right direction, they usually cannot be achieved quickly after a disaster. However, the national response framework only accommodates short-term recovery efforts, not long-term recovery [23]. This means that over time, governmental and organizational interest in the community’s wellbeing may decline. Correspondingly, resources, assets, and capabilities present during the response phase may diminish or leave the community if the impacted community is not equipped to recover in the long-term. To ensure that disaster-impacted communities go on to long-term recovery and ultimately become self-sufficient, it is crucial that public health agencies, especially those involved in the response phase of the disaster, fully engage these communities early by partnering with community stakeholders, representatives and assets.

This paper will demonstrate how Graniteville, a chemical disaster community, was effectively and successfully engaged during the disaster response and recovery phase and the role that continued community engagement and public health action has played in the community’s long term recovery and development of supplemental community-based participatory research (CBPR).

## 3. Methods

### 3.1. Background: Chemical Disaster in Graniteville, South Carolina

Graniteville, a small textile town of ~7,000 residents in South Carolina, experienced a chlorine spill on 6 January 2005. At 2:49 am, a freight train travelling at 49 mph collided with a parked train after the track was inadvertently switched onto an industrial spur. This collision resulted in its immediate derailment and the puncture of one of its three chlorine tank cars. An estimated 60 tons of liquid chlorine was released which quickly vaporized, producing a thick cloud of chlorine gas that spread throughout the town. Emergency services were activated and they responded promptly. Afterwards, a mass evacuation of Graniteville residents within a one mile perimeter was ordered to protect them from possible spills from the other two damaged, but unruptured chlorine tanks. Area schools and businesses in the evacuation zone were closed and some residents were not allowed to return to their homes for up to two weeks. Given the large amount of chlorine releases and the adverse health outcomes (mortality, morbidity, and hospitalizations), this was one of the worst exposures to chlorine gas by the general US population [24]. It resulted in the largest number of chlorine-related hospitalizations in recent times [24].

The chlorine spill occurred adjacent to the Avondale Textile Mill where about 180 people were working during the night shift. The incident resulted in nine immediate deaths, 72 hospitalizations for acute health effects from chlorine inhalation, and more than 840 people seeking medical attention. The consequences of the spill were far-ranging and severe, particularly because Graniteville was vulnerable from an economic and healthcare standpoint. Graniteville, being a medically underserved area (MUA) is characterized by a paucity of health infrastructure and healthcare providers as defined by HRSA [25]. Furthermore, Graniteville was an unincorporated town and was mainly dependent on Avondale Mill for the provision and upkeep of the community infrastructure, law enforcement and other civic services. Thus, besides the immediate effects of the chlorine spill, the subsequent permanent closure of the mill in 2006 was in itself a disaster because of Graniteville’s complete dependence on it.

### 3.2. Community Engagement Methods—Community-Based Participatory Service

Community engagement is the process of working collaboratively with and through groups of people affiliated by shared interests, geography, or proximity to address issues that affect their well-being [26]. It involves collaboration and partnerships and is an important tool to stimulate changes that improve the health of a community and its members [26]. A community-based participatory service (CBPS) approach was utilized by an external public health coalition of academic (University of South Carolina, Columbia, SC, USA) and state public health agencies (SCDHEC) in order to engage the Graniteville community following the chemical disaster [15]. CBPS, though similar to CBPR is somewhat distinct from it as it emphasizes the initial delivery of public health service, which may then be followed by public health research—different from CBPR where the primary goal is research which may then spur subsequent public health service or action [15].

The use of the CBPS approach was driven by the dire public health needs the Graniteville community faced immediately following the disaster as well as the ethics of conducting research on a community recently impacted by a disaster. In addition, this approach ensured community representation and participation in disaster response, relief and recovery efforts for public health planning while mitigating community distrust and building credibility of these efforts. It was also key to the success of long-term recovery efforts in Graniteville. A more detailed account of the CBPS activities used in the early disaster recovery period is presented elsewhere [15]. The following is an abbreviated account included as background to the longer-term public health recovery efforts.

After the initial response to the chemical disaster in Graniteville by first responders, public health workers from the environmental arm of the SCDHEC audited air monitoring and conducted safety inspections before the residents could reoccupy. During this process, SCDHEC established contact with a Graniteville community leader who presented some of the health and environmental concerns that were expressed by other community leaders and residents. These concerns included community distrust of response efforts, lack of community involvement in these efforts, health and environmental effects of chlorine exposure and the unavailability of appropriate medical care.

In the days following the disaster, and with assistance from SCDHEC, voluntary local leaders eventually came together to form the Graniteville Community Coalition (GCC). The principal purpose of the GCC was to assist the residents and community of Graniteville in recovering from the chlorine disaster by creating a self-sufficient Graniteville, improving developmental possibilities and offering health screening to residents directly impacted by the disaster. The GCC met with residents of the Graniteville community and came up with a list of environmental and health concerns raised by residents of Graniteville. SCDHEC staff reviewed these questions, answered them and developed a fact sheet detailing answers in an easily comprehensible fashion.

Subsequently, SCDHEC in collaboration with the GCC held a series of town hall public meetings and training workshops where its staff would meet with community members, and address their questions and issues. These meetings allowed residents to participate in the recovery of Graniteville by stating what they identified as pressing issues in the Graniteville community. For example, at these meetings, SCDHEC was able to solicit the opinions of community members on upcoming disaster recovery strategies. These meetings provided opportunities to mobilize the entire community and create a feeling of control and ownership in the recovery efforts. These meetings were also pivotal to building trust between the community and the external public health coalition. They created an atmosphere of trust and transparency while cultivating a relationship between SCDHEC and GCC that laid the foundation for a genuine and equitable partnership between SCDHEC and the greater Graniteville community. The GCC identified SCDHEC as a sincere partner in the recovery of Graniteville. As a result, the next steps for the recovery of Graniteville were made collaboratively between SCDHEC and GCC.

At the request of the community, SCDHEC performed further environmental sampling and monitoring to ensure environmental safety as well as to establish a community health tracking registry. The health branch of SCDHEC managed the health registry with the aim of identifying people directly involved in the disaster, providing links to medical resources for continued care, and a way to longitudinally track and follow-up with exposed individuals. Inclusion in the registry was voluntary. The environmental monitoring revealed that the air, water and soil were indeed safe with many residents finding these results very reassuring, though some were still doubtful. Despite this, SCDHEC’s extensive environmental health outreach and education efforts gradually began to dispel layers of distrust of response and recovery efforts.

In order to recruit people into the registry, other partnerships were formed with diverse local assets such as schools, businesses, faith-based organizations, and the University of South Carolina-Aiken (a local academic institution). This partnership helped to boost the registry’s credibility, increase its acceptability, and spread the word about the confidentiality of information collected, and its utility in tracking health outcomes long-term. Following the establishment of these partnerships, door-to-door visits were made and a local telephone line was opened, all efforts to recruit residents to participate in the registry. Some of the telephone operators were bilingual to cater to non-English speakers and most grew up in the Graniteville community and were long-term residents. Employing telephone operators from the impacted community was deliberate and important. By doing this, we built on existing community assets and capacity as the operators could serve as informal conduits for information about the goals of the registry while out in the community. The telephone operators were also in a better position to empathize with callers.

In August 2005, health screenings commenced on a rotating schedule at two area churches (one predominantly black, one predominantly white), and one local medical clinic. These health screenings were held over a period of 10 weeks to identify individuals who may have developed medical problems associated with exposure to chlorine or traumatic stress associated with the disaster. These screenings included vital signs assessment, medical and exposure histories, psychosocial health questionnaires, pulmonary function and reactivity tests, and evaluation of an air inflammation indicator.

Results of these screenings were reviewed with each individual and recommendations were made for follow-up care and where applicable, these individuals were referred to available health and social assistance resources in close proximity to the community. This was especially important given Graniteville’s status as a medically underserved community prior to the disaster. Due to the success of this initial set of health screenings, additional resources were granted for a second round of health screenings in 2007 from both the county and state totaling over $550,000.

The decision to site health screenings at these churches was done intentionally, to accommodate the diversity, culture and norms of the community, a key strategy in community engagement. A local behavioral health clinic was also used as a site because, as an existing asset within the community, residents were more familiar with it and more likely to trust screenings held there. Health screenings were staffed by volunteers from the community and SCDHEC contractors. All volunteers/contractors complied with SCDHEC’s confidentiality agreement and were health insurance portability and accountability act (HIPAA) trained. Like the telephone operators, volunteers were intentionally chosen from the community as part of engagement efforts and utilizing community assets, in this case, their experiences and skills. Integral to the process of community engagement in Graniteville described above was establishing partnerships, trust, building on existing community capacity and a consistent community presence by SCDHEC officials and its academic partners. These practices were instrumental in gaining acceptance, alleviating community concerns, enhancing community empowerment, and improving the likelihood of success of initial and long-term recovery efforts.

### 3.3. Transition from CBPS and Public Health Practice to Research

Our public health practice transitioned to public health research approximately three years after the disaster. The decision to transition was made in conjunction with the GCC and it commenced after the immediate and short-term relief and public health needs of the Graniteville community were met. It was important that the decision to commence research was made with the support of the GCC in order to establish our credibility and quell any suspicion.

The research agenda was driven by the concerns of residents who were concerned about the long-term health risks associated with chlorine exposure and the effect of the disaster on their overall quality of life. The goal of our public health research was to examine the health impact of the chlorine exposure on residents (adults and children) and millworkers in Graniteville. We utilized data from the health registry, pulmonary function tests, health screenings, and hospital records and examined the immediate and long-term effects of chlorine exposure on pulmonary function among adults, children, and millworkers. The community was also concerned about the mental health impact of the disaster. At the request of the community, we evaluated mental health and posttraumatic stress disorders among Graniteville residents. Our other research focused on the long-term impact of a disaster on health system response and performance and developing a framework for triage models in addressing a post-disaster secondary surge.

Our community engagement was vital to the success of our research. The GCC and representatives of Graniteville drove the focus of the research. We engaged community assets, both physical and human, during the public health practice and research phases. We trained and hired residents of Graniteville as data collectors, telephone operators, and health screening and medical personnel. We also utilized community assets such as the area churches and the local medical centers as health screening sites. Furthermore, we ensured that the residents of Graniteville understood that they had a voice and we were willing to listen to that voice. We first established a community advisory board (CAB) in 2009 to provide feedback on research activities, particularly the development of research questions that the partnership should focus on. In addition, we conducted focused interviews with residents and collected data on community concerns using surveys. We trained community members in photography and used Photovoice as a tool for community members to project themselves and their communities [27,28,29,30,31,32]. Finally, the GCC was intricately involved in the grant submission and grant writing process. Grant proposals were written with the members of the GCC providing substantial input and sanctioning the goals and objectives of these research proposals.

Institutional review board (IRB) approval for our work in Graniteville was obtained from the University of South Carolina, Columbia and the South Carolina Department of Health and Environmental Control (SCDHEC) in two phases. The initial approval was deemed public health practice and not research by both IRBs because the activities were aimed primarily at improving the health of the impacted population. These activities included the health screenings, the establishment and inclusion of participants into the registry, the public health meetings, public health workshops, and the linkage of participants into medical and psychological care. However, IRB approval was again sought from the University of South Carolina, Columbia, when our work transitioned from public health practice to research.

## 4. Results

We present the results of our community engagement by demonstrating how the initial public health efforts have produced a sustainable impact and facilitated research efforts that will further the overall recovery of Graniteville. The health registry that was started shortly after the chemical disaster has evolved into a longitudinal cohort study. This evolution was in large part to community buy-in and the continued presence of the academic partners in the community almost a decade after the disaster. Community members understood that their data would not be used without their permission and when used, it would be used to advance their health. They were therefore willing to continually get screened and obtain routine pulmonary function tests to evaluate their pulmonary function and health at the new Graniteville Recovery and Chlorine Epidemiology (GRACE) study center. Current grant funding has helped to build a state-of-the-art pulmonary diagnostic laboratory at the GRACE study center. The Graniteville community will be able to use the center in the coming years to assess their long-term pulmonary health post-disaster.

Our community engagement activities led to other efforts to determine the long-term impacts of the chemical spill on the quality of life and healthcare services access by Graniteville residents’ from the perspective of community residents and area healthcare providers. We achieved this using Photovoice [27,28,29,30,31,32]. Photovoice is a qualitative method of inquiry that purports that a photograph can provide the researcher with valuable insights into the cultural practices and lived experiences of individuals and communities [27,28,29,30,31,32]. The method helps individuals, especially individuals in populations that might otherwise not have a voice in policy development or decision-making, to document their lived experiences through the use of photography.

Other results of our community engagement activities include improvements to Graniteville’s poor physical infrastructure such as water and sewer infrastructure. With the mill’s closure, the infrastructure that was previously managed by the mill required repairs. In partnership with the GCC, we obtained a grant that funded the installation of water delivery infrastructure in order to provide clean and potable water to community residents. Understanding that long-term chlorine exposure was another major concern of the GCC and Graniteville residents, we sought to limit and remediate the long-term effects of chlorine exposure. Our collaborative partnership was awarded grant funding to assist with educating the community on emergency preparedness, and the effects of chlorine on their health and the environment. Other grants supported the retraining of former millworkers and others in Hazardous Waste Operations and Emergency Response (HAZWOPER) training for jobs at the local Savannah River Site Nuclear Facility in Aiken, South Carolina. In addition, members of the collaborative partnership received three brownfield redevelopment grants to assess the residual hazards in the abandoned mill buildings and remediate them so that they could bring in new businesses and industries.

Lastly, we received grant funding to study the initial triage of more than 800 people seeking medical care at local hospitals. Victims of chlorine exposure may experience difficulty breathing or shortness of breath immediately if high concentrations of chlorine gas are inhaled, or signs/symptoms may be delayed if low concentrations of chlorine gas are inhaled [24]. Thus, rapid assessment must be conducted upon exposure to chlorine. To mitigate the “surge” of such large numbers of casualties with limited healthcare resources, responders used triage assessments and relied on tools such as pulse oximetry (*i.e.*, a physiological measure for determining the amount [percent] of oxygen being carried by red blood cells) [33] and their clinical judgment to triage patients [34,35,36]. It is clear that the triage approaches used in the Graniteville disaster, although extemporaneous and diverse, worked reasonably well because only one hospitalized patient later died [15,24]. Likewise, the health screenings and the healthcare referral resources provided by the public health coalition were responsible for buffering the effect of the secondary surge after the disaster by providing alternate and effective sources of healthcare [37]. In total, the use of CBPS in the public health recovery of Graniteville has contributed to several million dollars in grant funding for public health affiliated services and the development of community infrastructure services. The town of Graniteville now has a state-of-the-art pulmonary diagnostic laboratory and a health resource center which is a foundation for current and future research activities. By focusing on helping the disaster community first, the Graniteville CBPS team has laid a powerful foundation for both current and future CBPR research opportunities that can translate results to public health action.

## 5. Discussion

Current literature suggests that many emergency and disaster response agencies fail to engage and acknowledge the role local citizen-led groups may play in disaster response resulting in a failure to adopt collaborative disaster response efforts [38]. Furthermore, the emphasis on research rather than public health service in the immediate post-disaster period [39] may reinforce stereotypes of researchers as putting professional interests ahead of the community’s interests. They may also raise the issue of ethics in conducting immediate research on vulnerable disaster populations, despite the implications of the study’s findings. On the other hand, engaging local groups with an initial focus on public health relief and recovery efforts, and then performing research in response to the community’s desire is a more ethical and sensitive approach, resulting in greater success.

In the case of Graniteville, the adoption of CBPS was vital to the success of the public health coalition partnership with the GCC. SCDHEC’s public health service provided an opportunity to identify community concerns and liaise with community leaders. Besides this, soliciting their input on various issues was important in building a sincere and trusting working relationship. Keeping all community partners informed of the progress of the public health efforts as well as involving them in the decision-making process helped initiate and institutionalize recovery efforts within the community. Town hall meetings and use of the community advisory board also provided an opportunity to bring all community groups together in order to address the common disaster that had befallen them and solicit buy-in for the disaster recovery efforts.

Other factors integral to laying the foundation for long-term recovery included the development of partnerships that relied on local assets, infrastructure and capacity. Local meeting and office space, staff and volunteers were recruited from the community to participate in community recovery efforts. Acts like this were crucial in securing community interest and engagement. They suggested to the community that we valued their knowledge, resources, skills and personnel and were committed to remaining in Graniteville during all phases of recovery. Recruiting staff and volunteers from the community served to empower impacted residents and make them conscious of the fact that they were contributing to the Graniteville recovery.

Similarly, utilizing staff from the community with contextual expertise in community health screenings and administrative duties of the recovery efforts helped to sustain knowledge about the disaster and value of collective efficacy [40]. It also provided an opportunity for these same individuals to talk to skeptical residents of Graniteville about the importance of the recovery efforts in Graniteville. These actions were all intentionally performed with the goal of building on and improving community assets to implement long-term solutions such as economic and community development initiatives, all of which are currently underway. The partnership with the academic and government agencies has also provided the GCC with the expertise it lacked, for example in seeking and obtaining other grant funding to further support recovery efforts.

## 6. Conclusions

Too often the focus of scientists within the allied public health fields is too narrowly on “how” to do good science in disaster populations rather than on “when”. We have shown that good public health research can be achieved in disaster communities, but preferably after the public health recovery and service needs are first collectively addressed. Disaster populations can teach us how to prevent future disaster, about the health effects from chemical exposures, and how to improve public health and medical infrastructure disaster preparedness. However, such research must first follow meaningful public health actions through community engagement.

Community engagement remains one of the most important tools in assisting chemical disaster populations, especially for long-term recovery. Such actions help to mitigate the adverse impacts of the disaster and provide technical know-how and support to build up capacity following the chemical disaster. They also serve to promote and encourage emergent collective behavior that addresses community-identified problems, cultivate greater community trust, and lay a strong foundation for subsequent research.

Community engagement can also form the basis for sustainability and encourage future community-collaborative health partnerships between impacted communities, government agencies, academic institutions, and other stakeholder groups. It may not only address the immediate effects of the disaster but through sustainability initiate long-term recovery, and form the basis upon which secondary effects of the disaster can be identified (e.g., long-term exposure to chlorine gas in Graniteville’s case) and alleviated promptly and effectively. The case of Graniteville successfully highlights the viable simultaneous interplay between a disaster response, disaster recovery and community engagement, all premised upon the principles of CBPS [15].
